# Repeatability and variation of region-of-interest methods using quantitative diffusion tensor MR imaging of the brain

**DOI:** 10.1186/1471-2342-12-30

**Published:** 2012-10-11

**Authors:** Ullamari Hakulinen, Antti Brander, Pertti Ryymin, Juha Öhman, Seppo Soimakallio, Mika Helminen, Prasun Dastidar, Hannu Eskola

**Affiliations:** 1 Department of Radiology, Medical Imaging Center of Pirkanmaa Hospital District, Teiskontie 35 PL 2000, 33521, Tampere, Finland; 2 Department of Biomedical Engineering, Tampere University of Technology, Tampere, Finland; 3 Department of Neurosurgery, Tampere University Hospital, Tampere, Finland; 4 Tampere Medical School, University of Tampere, Tampere, Finland; 5 Science Center of Pirkanmaa Hospital District, Tampere, Finland

## Abstract

**Background:**

Diffusion tensor imaging (DTI) is increasingly used in various diseases as a clinical tool for assessing the integrity of the brain’s white matter. Reduced fractional anisotropy (FA) and an increased apparent diffusion coefficient (ADC) are nonspecific findings in most pathological processes affecting the brain’s parenchyma. At present, there is no gold standard for validating diffusion measures, which are dependent on the scanning protocols, methods of the softwares and observers. Therefore, the normal variation and repeatability effects on commonly-derived measures should be carefully examined.

**Methods:**

Thirty healthy volunteers (mean age 37.8 years, SD 11.4) underwent DTI of the brain with 3T MRI. Region-of-interest (ROI) -based measurements were calculated at eleven anatomical locations in the pyramidal tracts, corpus callosum and frontobasal area. Two ROI-based methods, the circular method (CM) and the freehand method (FM), were compared. Both methods were also compared by performing measurements on a DTI phantom. The intra- and inter-observer variability (coefficient of variation, or CV%) and repeatability (intra-class correlation coefficient, or ICC) were assessed for FA and ADC values obtained using both ROI methods.

**Results:**

The mean FA values for all of the regions were 0.663 with the CM and 0.621 with the FM. For both methods, the FA was highest in the splenium of the corpus callosum. The mean ADC value was 0.727 ×10^-3^ mm^2^/s with the CM and 0.747 ×10^-3^ mm^2^/s with the FM, and both methods found the ADC to be lowest in the corona radiata. The CV percentages of the derived measures were < 13% with the CM and < 10% with the FM. In most of the regions, the ICCs were excellent or moderate for both methods. With the CM, the highest ICC for FA was in the posterior limb of the internal capsule (0.90), and with the FM, it was in the corona radiata (0.86). For ADC, the highest ICC was found in the genu of the corpus callosum (0.93) with the CM and in the uncinate fasciculus (0.92) with FM.

**Conclusions:**

With both ROI-based methods variability was low and repeatability was moderate. The circular method gave higher repeatability, but variation was slightly lower using the freehand method. The circular method can be recommended for the posterior limb of the internal capsule and splenium of the corpus callosum, and the freehand method for the corona radiata.

## Background

Diffusion tensor imaging (DTI) is an MRI technique that has been increasingly used as both a scientific and a clinical tool in the past decade [1]. DTI is based on the diffusion characteristics of water molecules and it enables investigation of the architecture of the biological environment [2] that cannot be seen by conventional magnetic resonance MRI techniques. In the brain area, DTI is used for visualizing and characterizing white matter tracts in which water diffusion follows the direction of fibers. The diffusion metrics such as fractional anisotropy (FA) and apparent diffusion coefficient (ADC) are often used in the diffusion analysis. FA is a measure of the degree of diffusion anisotropy, and ADC describes the average diffusion [3]. Decreased FA values and increased ADC values are related to the disruption of the tissue microstructure, including the axons in white matter tracts [4].

The diffusion measures have been used to evaluate the integrity of white matter tracts in pathological conditions [5-7] and in healthy brains [8-11]. FA and ADC changes have been found in several white matter diseases [12-16], but it is known that age affects both FA and ADC values, and small changes occur across the lifespan and even in different ways with men and women [17].

The quantitative DTI is still a relatively new method, and therefore it is essential to be aware of the variables and limitations relating to technique. For example, low signal-to-noise ratio (SNR), many artifacts [18-20] as well as partial volume effects impact on derived measures. The diffusion measures are also dependent on angular resolution and spatial resolutions [21] which affect the particular values of FA. In order to interpret the findings correctly observers need to realize these factors.

Region-of-interest (ROI) -based [1,11,22] and voxel-based methods [23,24] are the most commonly used quantitative approaches. The ROI-based method has been available for a longer time, and therefore, most clinical softwares include only this approach. In this method, the measurements are performed on the original slices, thus avoiding post-processing calculation errors, but the method suffers from the lack of normal values, and relatively low repeatability [22] and high variability [25]. Voxel-based methods are increasingly used in the research and they are more automated and are not dependent on the observer, but these methods require inter-subject registration and image smoothing [26]. One of the recent methods "tract-based spatial statistics" (TBSS) is fully automated, simple to use and investigates the whole brain. It aims to solve voxel-based statistics across subjects on the skeleton-space FA data.

An alternative technique to ROI measurements is fiber tracking (tractography). With this method, the FA and ADC values are averaged for the fiber bundles. Most commonly, tractography is based on convential DTI, but it suffers from difficulties with complex fiber architecture like crossing fiber tracts [27,28]. New techniques, such as high angular resolution diffusion imaging (HARDI), are able to solve these difficulties by measuring the diffusion attenuation in more angular directions. HARDI reconstruction techniques, such as Q-ball imaging [29], are particularly useful for reproducing complex fiber geometries and can lead to an SNR even lower than that of DTI [28].

Although various other methods have been suggested for neuroradiological quantifications, we have applied the ROI-based methods in this study. This is because these methods have wide availability and easiness to use in individual patients. The aim of our study was to evaluate these quantitative methods and to give preferences for the two ROI approaches. The analysis was based on intra- and inter-observer variation and repeatability. According to the medical literature no other studies with comparison of two specific DTI-based ROI methods have been measured in normal adults.

## Methods

### Subjects

Thirty healthy adults were scanned with a 3T Siemens Trio (Siemens Healthcare, Erlangen, Germany). The volunteer group consisted of 21 women and 9 men with an age range of 18 – 60 years and a mean age of 37.8 years [11]. MRI scans were performed during the autumn of 2008. The criteria for selecting the control group were age, sex and intelligence matching with patients enrolled in a mild traumatic brain injury study [30]. The volunteers included hospital staff and their relatives with no history of neurological or psychiatric diseases. The ethics committee of the hospital approved the study, and an informed consent was received from each volunteer.

### DTI phantom

The DTI phantom consisted of winding polyamide fibers (polyfil, 15-μm fibers, 50 dtex, Filamentgarn TYPE 611, Trevira GmbH, Bobingen, Germany) around an acrylic glass spindle [31]. The fluid portion consisted of an aqueous sodium chloride solution and distilled water (83 g NaCl per kilogram of water). The concentration of sodium chloride was matched to the susceptibility of the fluid and fibers [32]. According to the information provided by the manufacturer, the reference values were FA = 0*.*820 and ADC = 0*.*832 ×10^-3^ mm^2^/s.

### MRI acquisition

The MRI protocol included sagittal T1-weighted 3D IR-prepared gradient echo, axial T2-weighted turbo spin echo, conventional axial and high-resolution sagittal FLAIR (Fluid Attenuation Inversion Recovery), axial T2*-weighted, and axial SWI (Susceptibility Weighted Imaging) series. The DTI data were collected by a single-shot, spin echo-based, and diffusion-weighted echo planar imaging sequence. The parameters for the DTI sequence were TR 5144 ms, TE 92 ms, FOV 230 mm, matrix 128 × 128, 3 averages, slice/gap 3.0/0.9 mm, voxel dimension 1.8×1.8×3.0 mm, b-factor 0 and 1000 s/mm^2^, and 20 diffusion gradient orientations. A 12-channel head matrix coil was used. The DTI phantom was imaged using the same protocol and equipment as with the volunteers. Two 7-cm loop coils were used with the 12-channel head matrix coil to increase the SNR of the measurement.

### Data analysis

Multi directional diffusion data was first analysed visually for distortions and artifacts. Eddy current distortion was qualitatively estimated by drawing the brain contours to b_0_ images, and copying them to diffusion weighted images. We did not find significant eddy current distortions due to diffusion gradients.

Two observers, a physicist (UH) and a neuroradiologist (AB), separately performed the volunteer ROI measurements on a workstation using the commercially available software Neuro3D (Siemens Healthcare, Malvern, USA). With the circular (CM) and the freehand method (FM), ROIs were manually placed on axial images of the color-coded FA maps [33] and automatically transferred on the non-diffusion-weighted b_0_ images and ADC maps. The ROIs of the corpus callosum were drawn onto the median-line sagittal images. The ROIs were centered in the region using color-coded directions while taking care to avoid border areas, such as areas overlapping with cerebrospinal fluid spaces and neighboring tracts. The size of the ROI was chosen by using the subject´s own anatomical knowledge of regions. The measurements were similar to those performed in ordinary clinical conditions; for example, the levels of the slices were chosen each time the measurement was performed. The time between the first and repeat measurements was at least four weeks.

**Figure 1:**
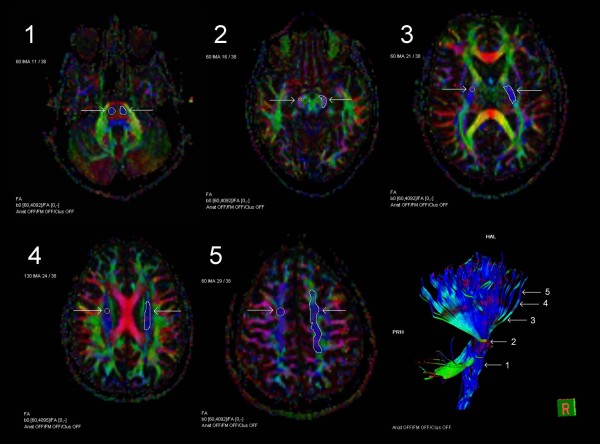
**ROIs in the pyramidal tracts.** Axial FA color maps with the circular and freehand ROIs (region-of-interest), and tractography of the pyramidal tract with five locations of the measurements: basal pons [1], cerebral peduncle [2], posterior limb of the internal capsule [3], corona radiata [4] and centrum semiovale [5].

**Figure 2:**
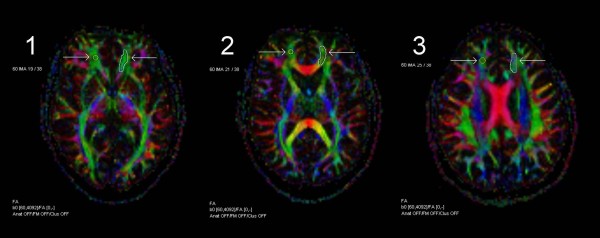
**ROIs in the frontobasal area.** ROI placements on axial FA color maps with circular and freehand methods in the frontobasal area: uncinate fasciculus [1], forceps minor [2] and anterior corona radiata [3].

**Figure 3:**
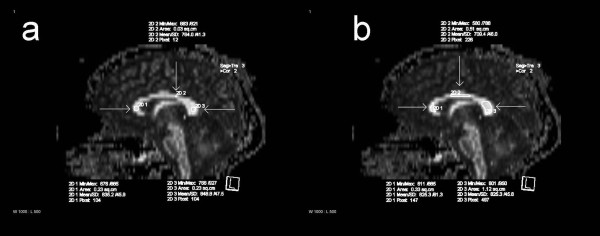
**ROIs in the corpus callosum.** ROI placements on sagittal FA maps in the corpus callosum with the circular (**a**) and freehand (**b**) methods: genu, body and splenium.

The FA and ADC values were measured in eleven separate regions. The ROIs for the pyramidal tracts included the basal pons, cerebral peduncle, posterior limb of the internal capsule, corona radiata and centrum semiovale (Figure 1). In the frontobasal area, the regions of interest were the uncinate fasciculus, forceps minor and anterior corona radiata (Figure 2). In the corpus callosum, the ROIs included the genu, body and splenium (Figure 3).

**Figure 4:**
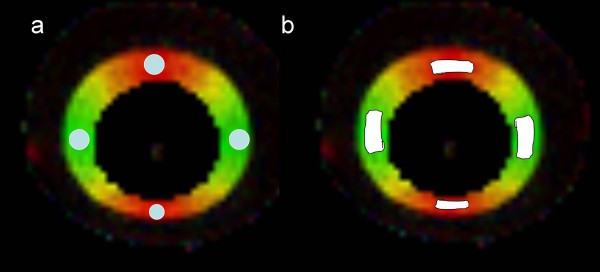
**ROIs in the phantom images.** ROI placements on axial FA color maps in the phantom with the circular (**a**) and freehand (**b**) methods.

With the phantom measurements, the ROIs were placed in four different regions (Figure 4).

Signal-to-Noise Ratio (SNR) was determined according the NEMA Standards 1–2008, including the following expression for SNR:

$$SNR=\frac{S}{\text{image\_noise}}$$

where S = signal, and image noise is estimated with Rayleigh distribution:

$$\text{image}\_\text{noise}=\frac{SD}{0.66}$$

The SNR measurements were used from the images of three subjects. The ROI was placed on the left side of the each region of the b = 0 s/mm^2^ image. The standardized ROI of the background noise (9.7 cm^2^) was placed outside the anatomical structures. SNR measurements were also performed in the DTI phantom. These four measurements were placed at similar locations as the actual measurements.

### Statistical analyses

The statistical analyses were performed using the SPSS software package (SPSS, Chicago, IL). The normality of the distributions was tested using the Kolmogorov-Smirnov test. The regional mean values were calculated from the mean values of the right and left hemispheres (N = 30). The FA and ADC values of the left and right hemispheres were compared using paired t-tests with the two-tailed significance set at p < 0.05/11. It was used Bonferroni correction for 11 regions instead of 22, because FA and ADC are relative independent. The right and left hemisphere asymmetries were evaluated according to the formula (A) = (left-right)/ ((left+right)/2) [25]. The differences between repeat measurements and measurements made by two different ROI-methods were compared using the standard deviation of the differences. These d ± 2s limits for the difference are known as the 95% limits of agreement, and these limits can be displayed as horizontal lines. This graphical representation is called a Bland-Altman plot [34]. The coefficient of variation (CV%) was calculated according the following equation (with SD = standard deviation and d = mean):

$$CV\%=\frac{SD}{d}\cdot 100\%\text{.}$$

The intra– and inter-observer repeatability values were assessed using the averages of intra-class correlation coefficients (ICCs) with absolute agreement. The ICC values were considered to indicate excellent agreement if they were greater than 0.8 and substantial agreement if they were from 0.60 to 0.79 [35]. The absolute p values were also defined, the statistical significance of which was set to p < 0.05/11 with Bonferroni correction for 11 regions. The results of the DTI phantom were compared with the values supplied by the manufacturer using the equation (with MV = measured value and RV = reference value):

$$\text{Change}\%=\frac{MV-RV}{RV}\cdot 100\%$$

## Results

**Figure 5:**
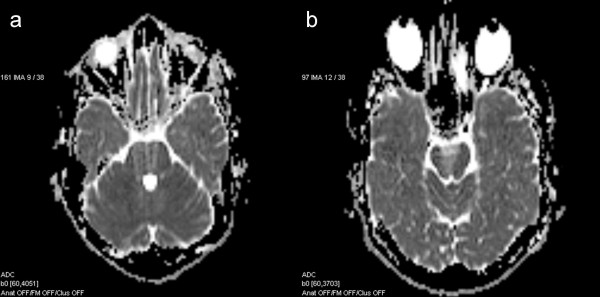
**Artifacts.** Axial ADC maps with the artifacts in the basal pons (**a**) and cerebral peduncle (**b**).

The preliminary results on the part of the pyramidal tract have been presented in our previous study [33], but in this study, the results are a part of a wider context. Using visual inspection the data quality was excellent in most cases, except in certain regions of the basal pons (Figure 5a), cerebral peduncle (Figure 5b) and the body of the corpus callosum, which had artifacts caused by air-filled cavities, pulsation or water containing tissues.

### Mean values for FA and ADC

The Kolmogorov–Smirnov test found that all mean values (N = 30) were normally distributed (p > 0.10). The intra-observer mean values, with the CM and the FM for FA and ADC, are shown in Table 1. The mean FA values were highest in the splenium of the corpus callosum with both methods; they were lowest in the corona radiata with CM and in the forceps minor with FM. The mean ADC values were highest in the body of the corpus callosum and lowest in the corona radiata with both methods. The values were 2.8% lower for FA and 0.4% higher for ADC with the FM compared to the CM.

**Table 1:** **The intra-observer regional mean FA and ADC (10**^**-3**^**mm**^**2**^**/s) values (mean ± sd) for the circular and freehand ROI methods (N = 30)**

		**Circular ROI**		**Freehand ROI**	
**Region**	**Parameter**	**1st meas.**	**2nd meas.**	**1st meas.**	**2nd meas.**
Basal pons	FA	0.653 ± 0.065	0.637 ± 0.070	0.573 ± 0.060	0.634 ± 0.054
	ADC	0.708 ± 0.052	0.709 ± 0.060	0.709 ± 0.044	0.705 ± 0.041
Cerebral peduncle	FA	0.853 ± 0.052	0.847 ± 0.049	0.779 ± 0.049	0.817 ± 0.041
	ADC	0.711 ± 0.057	0.710 ± 0.051	0.758 ± 0.058	0.728 ± 0.033
Internal capsule, post	FA	0.734 ± 0.043	0.737 ± 0.038	0.671 ± 0.042	0.714 ± 0.037
	ADC	0.685 ± 0.024^a^	0.679 ± 0.024	0.685 ± 0.015	0.688 ± 0.017
Corona radiata	FA	0.508 ± 0.053^b^	0.504 ± 0.050 ^c^	0.511 ± 0.049	0.524 ± 0.046 ^d^
	ADC	0.644 ± 0.027	0.648 ± 0.027	0.671 ± 0.025	0.663 ± 0.022
Centrum semiovale	FA	0.505 ± 0.053	0.516 ± 0.081	0.507 ± 0.047	0.558 ± 0.055
	ADC	0.688 ± 0.034	0.693 ± 0.042	0.698 ± 0.021	0.692 ± 0.022
Uncinate fasciculus	FA	0.542 ± 0.051	0.535 ± 0.064	0.501 ± 0.039 ^e^	0.552 ± 0.041
	ADC	0.773 ± 0.032	0.773 ± 0.032	0.774 ± 0.025	0.777 ± 0.032
Forceps minor	FA	0.539 ± 0.059	0.548 ± 0.074	0.466 ± 0.044 ^f^	0.511 ± 0.045
	ADC	0.764 ± 0.039	0.765 ± 0.031	0.761 ± 0.029	0.760 ± 0.027
Ant corona radiata	FA	0.551 ± 0.066	0.542 ± 0.073	0.472 ± 0.038	0.508 ± 0.043
	ADC	0.742 ± 0.027	0.759 ± 0.028	0.747 ± 0.027	0.750 ± 0.027
CC genu	FA	0.834 ± 0.041	0.826 ± 0.036	0.673 ± 0.049	0.810 ± 0.033
	ADC	0.758 ± 0.067	0.766 ± 0.051	0.858 ± 0.056	0.770 ± 0.049
CC body	FA	0.750 ± 0.048	0.723 ± 0.038	0.616 ± 0.054	0.683 ± 0.063
	ADC	0.755 ± 0.073	0.839 ± 0.084	0.896 ± 0.076	0.810 ± 0.090
CC splenium	FA	0.856 ± 0.055	0.855 ± 0.050	0.742 ± 0.051	0.836 ± 0.044
	ADC	0.713 ± 0.055	0.714 ± 0.040	0.792 ± 0.047	0.732 ± 0.042

Regions with significant differences between right and left cerebral hemispheres (p< 0.05/11):

^a^ Posterior limb of the internal capsule ADC (circular): a) right 0.699±0.028, left 0.671±0.037.

^b,c^ Corona radiata FA (circular): b) right 0.484±0.055, left 0.533±0.063, c) right 0.490±0.055, left 0.518±0.058.

^d^ Corona radiata FA (freehand): d) right 0.508±0.048, left 0.539±0.054.

^e^ Uncinate fasciculus FA (freehand): e) right 0.512±0.045, left 0.490±0.039.

^f^ Forceps minor FA (freehand): f) right 0.481±0.046, left 0.452 ±0.048.

Statistically significant differences were found between right and left hemisphere in the four regions. These regions were the posterior of the internal capsule with the CM, uncinate fasciculus and forceps minor with the FM, and the corona radiata with both methods.

Table 2 shows the observer 2 results for the mean FA and ADC values. Using CM, observer 2 had 1.0% higher average FA and 0.6% higher average ADC mean values than did observer 1. Using FM, observer 2 had 2.9% lower FA values and 2.4% higher ADC values than observer 1.

**Table 2:** **Measurements by observer 2 (mean ± sd), inter-observer repeatability (Obs. 1: 2. meas.) and variation (N = 30) for FA and ADC (10**^**-3**^**mm**^**2**^**/s)**

		**Circular ROI**				**Freehand ROI**			
**Region**	**Parameter**	**Mean ± SD**	**ICC**	**p**	**CV(%)**	**Mean ± SD**	**ICC**	**p**	**CV(%)**
Basal pons	FA	0.705 ± 0.044	0.44	0.004*	8.6	0.621 ± 0.061	0.73	< 0.001*	9.2
	ADC	0.726 ± 0.069	0.24	0.226	8.9	0.730 ± 0.080	0.01	0.487	8.4
Cerebral peduncle	FA	0.850 ± 0.035	0.62	0.006	4.9	0.788 ± 0.062	0.14	0.319	6.4
	ADC	0.706 ± 0.048	0.56	0.018	7.0	0.772 ± 0.070	0.49	0.008	6.8
Internal capsule, post	FA	0.767 ± 0.046	0.67	< 0.001*	5.6	0.728 ± 0.036	0.80	< 0.001*	5.0
	ADC	0.696 ± 0.034	0.63	0.001*	4.2	0.693 ± 0.021	0.84	< 0.001*	2.8
Corona radiata	FA	0.508 ± 0.038	0.54	0.023	8.6	0.515 ± 0.047	0.78	< 0.001*	9.0
	ADC	0.648 ± 0.030	0.64	0.005	4.5	0.665 ± 0.025	0.93	< 0.001*	3.6
Centrum semiovale	FA	0.501 ± 0.067	0.64	0.004*	14.5	0.509 ± 0.051	0.47	0.007	9.9
	ADC	0.700 ± 0.042	0.75	< 0.001*	6.0	0.693 ± 0.024	0.86	< 0.001*	3.3
Uncinate fasciculus	FA	0.529 ± 0.060	0.62	0.007	11.7	0.517 ± 0.050	0.74	< 0.001*	8.5
	ADC	0.779 ± 0.036	0.55	0.018	4.4	0.768 ± 0.025	0.84	< 0.001*	3.7
Forceps minor	FA	0.545 ± 0.057	0.64	0.004*	12.0	0.499 ± 0.051	0.83	< 0.001*	9.5
	ADC	0.762 ± 0.046	0.17	0.319	5.1	0.757 ± 0.029	0.88	< 0.001*	3.7
Ant corona radiata	FA	0.551 ± 0.073	0.84	< 0.001*	13.7	0.497 ± 0.040	0.86	< 0.001*	8.3
	ADC	0.754 ± 0.029	0.45	0.060	3.8	0.748 ± 0.024	0.82	< 0.001*	3.5
CC genu	FA	0.836 ± 0.034	0.80	< 0.001*	4.2	0.785 ± 0.051	0.51	0.013	5.3
	ADC	0.765 ± 0.044	0.80	< 0.001*	6.2	0.846 ± 0.069	0.37	0.012	7.3
CC body	FA	0.696 ± 0.057	0.39	0.066	6.7	0.654 ± 0.069	0.62	0.003*	9.8
	ADC	0.821 ± 0.164	0.61	0.008	15	0.862 ± 0.114	0.65	0.001*	12.2
CC splenium	FA	0.864 ± 0.047	0.90	< 0.001*	5.6	0.838 ± 0.043	0.88	< 0.001*	5.2
	ADC	0.717 ± 0.038	0.81	< 0.001*	5.4	0.739 ± 0.039	0.80	< 0.001*	5.5

* = the statistical significance p < 0.05/11 with Bonferroni correction for 11 regions.

ICC = intra-class correlation coefficient; CV = coefficient of variation.

### SNR analysis

The mean SNR value (± standard deviation) of b = 0 s/mm^2^ images for all regions in vivo measurements was 25.4 ± 3.9, for the pyramidal tract 25.3 ± 3.7, for the corpus callosum 25.4 ± 5.1 and for the frontobasal area 25.4 ± 2.6. The mean SNR of the four regions of the DTI phantom was 27.9 ± 6.0.

### Intra- and inter-observer variation

The intra-observer variation (CV%) results are shown in Table 3. Using CM, the CV percentage for FA was below 10% in 5 of 11 regions. Using FM, this percentage was below 10% in all of the regions. For ADC, the CV percentage was below 10% in all of the regions with both methods. The mean variation of the FA results was 9% with the CM and 8% with the FM. For ADC, the variation was 6% with the CM and 5% with the FM. For FA, the highest variation was in the centrum semiovale with the CM and the basal pons with the FM. The lowest variation was found in the genu of the corpus callosum with the CM and in the cerebral peduncle with the FM. For ADC, both methods had the highest variation in the body of the corpus callosum and the lowest in the posterior limb of the internal capsule.

**Table 3:** **The intra-observer repeatability and variability (N = 30) for FA and ADC (10-**^**3**^**mm2/s) (N = 30)**

		**Circular ROI**				**Freehand ROI**			
					**Mean diff. ± 2sd**				**Mean diff. ± 2sd**
Region	Parameter	ICC	p	CV (%)		ICC	p	CV (%)	
Basal pons	FA	0.74	< 0.001*	10.4	0.020 ± 0.093	0.63	< 0.001*	9.5	−0.061 ± 0.085
	ADC	0.83	< 0.001*	7.9	−0.010 ± 0.038	0.70	0.001*	6.0	0.004 ± 0.082
Cerebral peduncle	FA	0.74	< 0.001*	5.9	0.006 ± 0.092	0.42	0.025	5.6	−0.038 ± 0.101
	ADC	0.19	0.297	7.6	0.001 ± 0.145	0.29	0.141	6.1	0.030 ± 0.120
Internal capsule, post	FA	0.90	< 0.001*	5.5	−0.002 ± 0.049	0.66	< 0.001*	5.7	−0.043 ± 0.051
	ADC	0.76	< 0.001*	3.5	0.006 ± 0.042	0.78	< 0.001*	2.3	−0.002 ± 0.027
Corona radiata	FA	0.84	< 0.001*	10.2	0.005 ± 0.077	0.86	< 0.001*	9.2	−0.013 ± 0.063
	ADC	0.76	< 0.001*	4.2	−0.004 ± 0.048	0.86	< 0.001*	3.6	0.008 ± 0.030
Centrum semiovale	FA	0.48	0.041	13.1	−0.001 ± 0.160	0.26	0.121	9.5	−0.051 ± 0.128
	ADC	0.63	0.005	5.5	−0.005 ± 0.079	0.87	< 0.001*	3.1	0.005 ± 0.028
Uncinate fasciculus	FA	0.67	0.002*	10.7	0.007 ± 0.116	0.61	< 0.001*	7.6	−0.051 ± 0.051
	ADC	0.69	0.001*	4.1	0.000 ± 0.063	0.92	< 0.001*	3.7	−0.003 ± 0.031
Forceps minor	FA	0.76	< 0.001*	12.2	−0.009 ± 0.118	0.65	< 0.001*	9.1	−0.044 ± 0.067
	ADC	0.64	0.005	4.6	−0.001 ± 0.073	0.89	< 0.001*	3.7	0.001 ± 0.035
Ant corona radiata	FA	0.71	0.001*	12.8	0.009 ± 0.133	0.61	< 0.001*	8.3	−0.036 ± 0.073
	ADC	0.27	0.169	3.7	−0.017 ± 0.071	0.85	< 0.001*	3.7	−0.002 ± 0.040
CC genu	FA	0.67	0.002*	4.7	0.008 ± 0.078	0.15	0.002*	5.7	−0.137 ± 0.082
	ADC	0.93	< 0.001*	7.7	−0.008 ± 0.062	0.37	0.001*	6.5	0.089 ± 0.102
CC body	FA	0.23	0.203	5.8	0.027 ± 0.112	0.59	< 0.001*	9.0	−0.067 ± 0.091
	ADC	0.26	0.107	9.8	−0.085 ± 0.195	0.50	0.002*	9.8	0.087 ± 0.166
CC splenium	FA	0.89	< 0.001*	6.1	0.002 ± 0.066	0.27	0.006	6.0	−0.094 ± 0.100
	ADC	0.82	< 0.001*	6.6	−0.001 ± 0.075	0.34	0.015	5.9	0.060 ± 0.099

ICC = intra-class correlation coefficient; CV = coefficient of variation; Mean diff. ± 2sd = 95% limits of agreement, * = the statistical significance p < 0.05/11 with Bonferroni correction for 11 regions.

The inter-observer CV% was below 10% in most of the regions; for FA measured with the CM, 7 of 11 regions had a CV% below 10%, and the FA in all of the regions measured with the FM had a CV percentage below 10%. For ADC, 9 of 11 regions had a CV percentage below 10% with both methods. The mean variation of the FA results was 9% with the CM and 7% with the FM, and for ADC, the variation was 8% with the CM and 6% with the FM.

**Figure 6:**
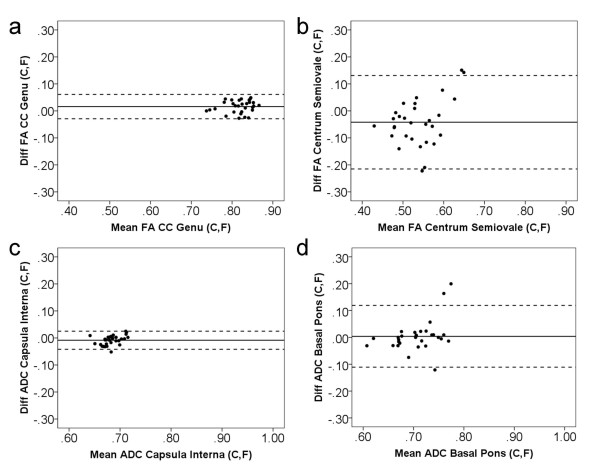
**The Bland–Altman plots**. Difference versus sum for FA (scaled 0–1) and ADC (10^-3^mm^2^/s) measured by circular (C) and freehand (F) methods. The Bland–Altman plots show maximum and minimum differences of regions with 95% limits of agreement (dotted lines): genu of the corpus callosum (FA) (**a**), centrum semiovale (FA) (**b**), posterior limb of the internal capsule (ADC) (**c**) and basal pons (ADC) (**d**).

The differences versus the sum (as measured by the FM and CM) for the FA and ADC values are shown in Figure 6. The Bland–Altman plots show minimum differences in the genu of the corpus callosum for FA and in the posterior limb of internal capsule for ADC and maximum differences in the centrum semiovale for FA and in the basal pons for ADC.

### Intra- and inter-observer repeatability

The intra-observer repeatability (ICC) results are shown in Table 3. For FA, the average ICC was higher with the CM (0.70) than with the FM (0.52); for ADC, however, the FM had a higher ICC (0.67) than the CM (0.61). The ICC results for FA were above 0.8 in 3 of 11 regions with the CM and in 1 of 11 regions with the FM. For ADC, the corresponding results were 3 of 11 with the CM and 5 of 11 with the FM. The best ICC for FA values was in the posterior limb of the internal capsule (0.90) with the CM and in the corona radiata (0.86) with the FM. For ADC, the best ICC was in the genu of the corpus callosum (0.93) with the CM and in the uncinate fasciculus (0.92) with the FM.

The best 95% limit for FA intra-observer agreement was found in the posterior limb of the internal capsule for the CM and in the uncinate fasciculus for the FM. The lowest level of agreement was in the centrum semiovale with both ROI methods. For ADC, both methods had the highest observed agreement in the posterior limb of internal capsule and the lowest in the body of the corpus callosum. With the FA and ADC measurements, statistically significant of the (p < 0.05/11) repeatability was found in 68% of the regions with the CM and 78% in the regions with the FM.

Table 2 shows the inter-observer repeatability (ICC) results. The ICC of the FA measurement was 0.65 with the CM and 0.67 with the FM, and for the ADC measurements it was 0.56 with the CM and 0.68 with the FM. For FA, the ICC results were 0.8 or above in 3 of 11 regions with the CM and in 4 of 11 regions with the FM. For ADC, the results were found in 2 of 11 regions with the CM and 7 of 11 regions with the FM. The best ICC results of the FA was found in the splenium of the corpus callosum with both methods (ICC for CM = 0.90 and ICC for FM = 0.88). For the ADC, the highest ICC was in the splenium of the corpus callosum (0.81) with the CM and in the corona radiata (0.93) with the FM.

### Phantom measurements

Using the CM method in phantom studies resulted in a mean FA value of 0.836 ± 0.017 (range 0.823 – 0.861) and a mean ADC value of 0.837 ± 0.016 ×10^-3^ mm^2^/s (range 0.823 – 0.857 ×10^-3^ mm^2^/s). Similarly, the FM results gave a mean FA value of 0.818 ± 0.010 (range 0.806 – 0.830) and a mean ADC value of 852 ± 0.009 ×10^-3^ mm^2^/s (range 0.841 – 0.862 ×10^-3^ mm^2^/s). For FA, the CM result differed from reference value CV% = 2.1, and for ADC it was CV% = 1.9. The corresponding FM results were CV% = 1.3 for FA and CV% = 1.0 for ADC. The SNR result of the phantom was presented above.

## Discussion

In this study, we investigated the mean values, variation and repeatability in an intra- and inter-observer study on MRI´s of 30 healthy volunteers using two methods based on circular and freehand regions of interests. The SNR and phantom measurements showed that the image quality was good and adequate for analysis purposes.

Regional variation of the FA and ADC absolute values was large, which has also been found in previous studies [11,33,36]. In our study the highest values were obtained in the corpus callosum, in concordance with Lee et al. 2009 and Brander et al. 2010. The high FA reflects the microstructure of corpus callosum in which the fibers are tightly packed and parallel to each other. The highest FA value within the pyramidal tract was found in the cerebral peduncle, which we have reported also in our previous study [33]. The results of the frontobasal regions were very close to each other in each region.

Brain asymmetries were noticed in some regions. They were found in the corona radiata for FA, and in the posterior limb of the internal capsule for ADC, which agree with previous findings [11]. In addition, asymmetries were found in the frontobasal area such as the uncinate fasciculus and forceps minor. Recently, Bonekamp et al. 2007 and Snook et al. 2005 have reported the existence of asymmetry in the centrum semiovale. Generally, the brain asymmetries have also been observed with other imaging modalities such as computed tomography (CT) [37].

The variation of the FA and ADC mean values were lower for the FM than for the CM. The results agree with the study by Bonecamp et al. 2009 and our earlier study, which included regions of the pyramidal tracts [33]. The repeatability was better with the CM than with the FM because the freehand ROIs included the borderzones of the tracts, which have lower FA than the central tracts. However, the results were highly dependent on the region.

The interregional variation was due to the location. It depended on the density of the tracts and also the artifacts, which were represented above. In addition, relative low spatial resolution effects especially in the small regions. Variation may also be caused by several factors such as noise level, gradient stability, motion and slice position between subjects [25]. The SNR of the b = 0 s/mm^2^ should be at least 20 in order to derive relatively reliable FA values [20]. In our study, the SNR was well above 20 in all regions expect basal pons (SNR = 19.2), and measured SNR are comparable to earlier study [38]. The variability of the intra-observer and inter-observer was relatively low at all regions. It was higher in the FA values than in the ADC values, as has been found in previous studies [1,11,19,25,39,40]. It is known that ADC values are homogeneous throughout the healthy brain, whereas FA values change depending on the location [41]. However, increased ADC variability was found in such regions as the cerebral peduncle and the corpus callosum, which is consistent with the study by Bonekamp [25]. In this study, the high variability in the body of the corpus callosum resulted from the effect of cerebrospinal fluid and the small ROI diameter. On average, the intra- and inter-observer variabilities were lower with the FM than with the CM.

According to 95% limits of agreement the differences between two methods was the smallest in the genu and splenium of the corpus callosum and in the posterior limb of the pyramidal tract for FA. The result can be explained by these regions being small, compact and usually without artifacts, so that the locations and size of the ROIs were almost the same. The differences were larger in the other regions because the ROI size between circle and freehand methods varied considerably. The CM represented a small sample area, whereas the freehand ROI covers the entire area of the measured tract. In the case of the basal pons and cerebral peduncle, the sources of variation were artifacts such as air-filled cavities, which affect the FM more than the CM.

The level of repeatability was moderate in most of the regions, as has been found in previous studies [11,22]. We found an excellent FA agreement in the posterior limb of the internal capsule and corona radiata, such as in the splenium of the corpus callosum, when using the CM [22]. The repeatability of the FA results was lower than that of the ADC results [19,25] because the partial volume effects and border areas had more effect on the FA values. Our results were consistent with the FM findings with the exception of the region of the body of the corpus callosum that was close to the cerebrospinal fluid. In general, the results were region-dependent. In most regions, repeatability was acceptable at the group level, but only few regions at the single-subject level.

The FA results of the DTI phantom showed more variation from the reference values when the CM was used than when the FM was used. In addition, the FA and ADC values were more variable for the FM than for the CM. Generally, the variability of both methods resembled that of the similar phantoms in previous studies [31]. The results of SNR were a bit higher for the phantom in comparison with in vivo measures. This difference is due to the fact that it we used two loop coils with the acquisition.

The repeatability of the results was decreased by the level being chosen separately each time, but this practice is a reality in the clinical environment. In addition, the examiner displayed learning effects, for example learning to avoid the artifacts and the border areas.

More investigations are needed to characterize different methods with a larger group of volunteers. These investigations should not concentrate only on ROI-based methods, but also studies comparing them to voxel-based methods would be important. These kinds of studies could also give rise to optimal combinations of different methods producing valuable new tools for the neuroradiologists.

## Conclusions

Both methods, the circular and freehand method, had low variability and moderate repeatability in most regions. Slightly less variation was found with the freehand method, but the repeatability was higher with the circular method. Based on our study, the circular method can be recommended for the posterior limb of the internal capsule and splenium of the corpus callosum, and the freehand method for the corona radiata.

## Competing interests

The authors assure that they have no competing interest.

## Authors' contributions

Guarantors of integrity of entire study, UH; study concepts/study design, all authors; data acquisition, PR, JÖ, SS, PD; data analysis/interpretation, UH, AB; manuscript drafting, UH; manuscript revision, all authors; approval of final version of submitted manuscript, all authors; literature research, UH; statistical analysis, UH, MH; and manuscript editing, UH, AB, PR, HE. All authors read and approved the final manuscript.

## Pre-publication history

The pre-publication history for this paper can be accessed here:

http://www.biomedcentral.com/1471-2342/12/30/prepub
